# Clinical Advances in Neuromuscular Diseases: Neurometabolic Disorders

**DOI:** 10.3390/muscles2030020

**Published:** 2023-07-17

**Authors:** Corrado Angelini, Daniela Tavian

**Affiliations:** 1 Department of Neurosciences, University of Padova, 35128 Padova, Italy; 2 Department of Psychology, Catholic University, 20123 Milan, Italy; daniela.tavian@unicatt.it

Metabolic myopathies are characterized by the dysfunction of several metabolic pathways that results in a deficiency of fuels required to generate energy for muscle contractions. The most common manifestations appear with neuromuscular symptoms due to impaired muscle development or functioning; however, in several diseases, there are multisystemic clinical manifestations. Metabolic disorders are characterized by the deficiency of essential metabolites for skeletal muscle, the heart, and its conduction apparatus, and the most common signs relate to neurological symptoms due to impaired muscle function, i.e., weakness and myoglobinuria, rigid spine, sleep dysfunction relating to nocturnal hypoventilation, fatigue, heart insufficiency, and impaired brain development or encephalopathy. Because most are rare disorders and present a low incidence and high fatality, metabolic disorders are, at their onset, significant for pediatric neurologists; however, while several metabolic defects can be diagnosed in adult patients, there are increasing numbers of infantile cases which, when treated, might transit from pediatric into adult services. 

The neurometabolic disorders of inborn error of metabolism with CNS involvement, i.e., vascular events, ischemic strokes, and aneurysms causing subarachnoid hemorrhage or lacunar encephalopathy, are therefore relevant to both pediatric and adult neurologists.

Regarding their presentation, the skeletal muscle signs are weakness, myalgia, exercise intolerance, and myoglobinuria, but cramps, macroglossia, dysarthria, and dysphagia are as frequent as heart or liver involvement, and brain abnormality. Weakness is the most common symptom of muscular metabolic disorders; the subsequent disability that patients experience depends on the specific muscle group involved and is usually prominent in the proximal lower girdle with hip weakness, but there are several cases with distal foot weakness, while proximal girdle shoulder weakness, axial muscle involvement such as neck muscle weakness, and rigid spine are less frequent.

To monitor such patients, it is important to measure muscle strength according to the MRC scale and skeletal muscle function via the six-minute walk test, GSGC scale, or other timed tests. Rather profound muscle weakness is a clinical sign appearing in several genetic muscle disorders due either to enzyme or sarcolemmal protein defects, i.e., late-onset Pompe disease, while asymmetric distal foot weakness is observed in neutral lipid storage disorder, due to PNPLA2 deficiency. 

The investigation of a suspected metabolic disorder can be difficult, but the presenting age, familiarity, and clinical history can be utilized in the approach. Characteristic examination findings, such as cramps, pain, muscle tenderness, and weakness, can help to narrow the differential diagnosis. For example, abnormal fatigue after exercise and subsequent weakness are common symptoms of abnormal glycogen storage due to the inability of the skeletal muscle or other tissues to use glycogen in the “glycogenosis” group, which now is composed of 15 glycogenosis subtypes.

Investigating rare conditions in metabolic cases, the investigations are likely to be expensive, difficult to perform, and potentially unrewarding, but making an accurate diagnosis is an important task. Therefore, the clinical domains regarding skeletal muscle strength and imaging and pulmonary functions should be extensively investigated, as there are rare treatable conditions, such as classic infantile Pompe disease (IOPD) or late-onset Pompe disease (LOPD) [1,2]. The two most common muscle glycogenosis are type 2 (Pompe) and type 5 (McArdle disease), which have either a proximal myalgia/weakness or intermittent fatigue as the main feature, while weakness appears in the late stages of the disease, but the onset might be characterized by exercise intolerance and rhabdomyolysis.

Regarding diagnosis and treatment, the signs and symptoms of metabolic disease resulting from myopathy are “Weakness”, “Hypotonia”, “Exercise intolerance”, and “Rhabdomyolysis”, in the muscle biopsy, characteristic abnormal pathologies, such as vacuoles or glycogen deposits, are found. However, such invasive investigation is nowadays preceded by muscle MRI imaging or even next-generation sequencing investigation. In general terms, weakness, hypotonia, exercise intolerance, and rhabdomyolysis are the most frequent symptoms associated with myopathies and are reported in 83%, 21%, and 6% of metabolic myopathies. Biomarkers in blood and urine are available and can be detected by dried blood spot (DBS) screening, which is useful both for glycogenosis type 2 and in the realm of fatty-acid disorders diagnosis by acyl-carnitines.

Fatty-acid oxidation (FAO) diseases are inborn errors of lipid metabolism that are caused by a deficiency of the enzymes needed to break down fatty acids, and this often results in the morphological presence of lipid droplets in skeletal muscle lipid storage myopathies (LSM). 

These autosomal recessive disorders, established on morphological grounds via the accumulation of lipid droplets (LDs) in the skeletal muscle, are due to several biochemical abnormalities. The four most frequent types of FAO diseases are due to mutations in genes encoding for specific enzymes of lipid metabolism, which might determine abnormal storage of LDs in muscle. They present myopathic symptoms and include the following entities: primary carnitine deficiency (PCD), riboflavin responsive multiple acyl-coenzyme A dehydrogenase deficiency (RR-MADD), neutral lipid storage disease with ichthyosis (NLSD-I), and neutral lipid storage disease with myopathy (NLSD-M) [3].

The study by DBS reveals a characteristic acylcarnitine profile in RR-MADD. The carnitine supplementation reverts cardiac dysfunction and muscle weakness in PCD, and riboflavin supplementation often results in a marked improvement in clinical weakness (50–100 mg 2–3 times daily) in RR-MADD. A diet poor in fat, with carnitine and MCT supplementation, avoiding long fasting periods, can also be utilized as well as medium-chain-triglyceride supplementation. Riboflavin therapy has resulted in benefits in more than 400 patients with ETFDH mutations, since most of them did improve their acute clinical and metabolic signs. Most of these patients are homozygotes or compound heterozygotes for missense mutations. 

The riboflavin/carnitine supplementation might, however, be partially effective or ineffective in some patients. A failure to respond to treatment is probably due to the presence of mutations that dramatically reduce ETFDH stability but could be because treatment was started late. To obtain a better response, riboflavin treatment should be started early in cases with late-onset RR-MADD to prevent severe metabolic crises. For Pompe disease, several important treatment milestones were achieved by ERT and the use of chaperons; after the first generation of al-glucosidase alpha was approved in 2006, a new version of recombinant aval-glucosidase alpha was approved in 2021/2022, and cipalglucosidase with miglustat in 2023, which are now available. There are therefore several new drugs to be tested, and their results in the long run will give interesting results. Nutritional and exercise protocols are also of benefit [4].

For LSM, a useful treatment is also based on controlled physical training to develop muscle oxidative mitochondrial capacity and programmed glucose intake following periods of exercise. Currently, many genetic therapies are being investigated at preclinical and clinical levels. However, these potential gene treatments are still variable and need immunosuppression, are gene-specific, and their delivery aims to treat diseases with only skeletal muscular manifestations. Unfortunately, for most metabolic disorders with the muscular phenotype, this is often complicated by CNS involvement, and this might contribute to life-threatening manifestations, such as respiratory center dysfunction, which requires urgent intervention and needs further research into methods by which to deliver gene therapy across the blood–brain barrier.

Editorial aim: we emphasize that in metabolic patients, extensive investigations are likely to be expensive, including muscle or brain MRI studies, which are difficult to perform and require specialized apparatus, such as the study of acyl-carnitines via DBS, and are potentially unrewarding. However, making an accurate diagnosis is an important challenge, particularly in glycogenosis type 2 and in primary or secondary states of carnitine deficiency due to RR-MADD. The use of new gadgets such as APPs or electronic bracelets for follow-up studies is an open field of investigation. We aim to further the field of metabolic myopathies by presenting new cases, instruments for disease monitoring, and treatment challenges, and we aim to identify future methods of achieving further progress in this field, both in basic and translational research.

## References

[1] Nascimbeni A.C., Fanin M., Masiero E., Angelini C., Sandri M. (2012). The role of autophagy in the pathogenesis of glycogen storage disease type II (GSDII). Cell Death Differ..

[2] Nascimbeni A.C., Fanin M., Tasca E., Angelini C., Sandri M. (2015). Impaired autophagy affects acid alpha-glucosidase processing and enzyme replacement therapy efficacy in late-onset glycogen storage disease type II. Neuropathol. Appl. Neurobiol..

[3] Missaglia S., Coleman R.A., Mordente A., Tavian D. (2019). Neutral Lipid Storage Diseases as Cellular Model to Study Lipid Droplet Function. Cells.

[4] Angelini C. (2021). Exercise and nutrition concomitant to enzyme replacement therapy are efficacious in adult Pompe patients:report from EPOC Consortium. Eur. J. Transl. Myol..

